# Diagnostic performance of artificial intelligence model for pneumonia from chest radiography

**DOI:** 10.1371/journal.pone.0249399

**Published:** 2021-04-15

**Authors:** TaeWoo Kwon, Sang Pyo Lee, Dongmin Kim, Jinseong Jang, Myungjae Lee, Shin Uk Kang, Heejin Kim, Keunyoung Oh, Jinhee On, Young Jae Kim, So Jeong Yun, Kwang Nam Jin, Eun Young Kim, Kwang Gi Kim

**Affiliations:** 1 JLK, Incorporated, Eonju-ro, Gangnam-gu, Seoul, South Korea; 2 Department of Internal Medicine, Gil Medical Center, Gachon University College of Medicine, Incheon, South Korea; 3 Korea National Tuberculosis Association (KNTA), Seoul, South Korea; 4 Department of Biomedical Engineering, Gachon University College of Medicine, Incheon, South Korea; 5 Department of Radiology, Seoul Metropolitan Government-Seoul National University Boramae Medical Center, Seoul, South Korea; 6 Department of Radiology, Gil Medical Center, Gachon University College of Medicine, Incheon, South Korea; Korea National University of Transportation, REPUBLIC OF KOREA

## Abstract

**Objective:**

The chest X-ray (CXR) is the most readily available and common imaging modality for the assessment of pneumonia. However, detecting pneumonia from chest radiography is a challenging task, even for experienced radiologists. An artificial intelligence (AI) model might help to diagnose pneumonia from CXR more quickly and accurately. We aim to develop an AI model for pneumonia from CXR images and to evaluate diagnostic performance with external dataset.

**Methods:**

To train the pneumonia model, a total of 157,016 CXR images from the National Institutes of Health (NIH) and the Korean National Tuberculosis Association (KNTA) were used (normal vs. pneumonia = 120,722 vs.36,294). An ensemble model of two neural networks with DenseNet classifies each CXR image into pneumonia or not. To test the accuracy of the models, a separate external dataset of pneumonia CXR images (n = 212) from a tertiary university hospital (Gachon University Gil Medical Center GUGMC, Incheon, South Korea) was used; the diagnosis of pneumonia was based on both the chest CT findings and clinical information, and the performance evaluated using the area under the receiver operating characteristic curve (AUC). Moreover, we tested the change of the AI probability score for pneumonia using the follow-up CXR images (7 days after the diagnosis of pneumonia, n = 100).

**Results:**

When the probability scores of the models that have a threshold of 0.5 for pneumonia, two models (models 1 and 4) having different pre-processing parameters on the histogram equalization distribution showed best AUC performances of 0.973 and 0.960, respectively. As expected, the ensemble model of these two models performed better than each of the classification models with 0.983 AUC. Furthermore, the AI probability score change for pneumonia showed a significant difference between improved cases and aggravated cases (Δ = -0.06 ± 0.14 vs. 0.06 ± 0.09, for 85 improved cases and 15 aggravated cases, respectively, *P* = 0.001) for CXR taken as a 7-day follow-up.

**Conclusions:**

The ensemble model combined two different classification models for pneumonia that performed at 0.983 AUC for an external test dataset from a completely different data source. Furthermore, AI probability scores showed significant changes between cases of different clinical prognosis, which suggest the possibility of increased efficiency and performance of the CXR reading at the diagnosis and follow-up evaluation for pneumonia.

## Introduction

Pneumonia is a contagious disease characterized by acute lower respiratory infection, usually caused by viruses or bacteria and less commonly, other microorganisms [1]. Pneumonia affects approximately 450 million people (7% of the population) globally, results in approximately 4 million deaths each year [2]. Typical symptoms include a combination of productive or dry cough, chest pain, fever, and breathing difficulties [3, 4]. The presentation might be atypical since it is influenced by patient age, comorbid conditions, lifestyle factors, and causative pathogens. Furthermore, because pneumonia shares many symptoms with other conditions such as the common cold, bronchitis, and asthma, the diagnosis is difficult with only symptoms and physical examination. CXR remains the most readily available and common imaging modality for the assessment of pneumonia [5, 6]. Radiologically, CXR presentations of pneumonia can be classified as lobar pneumonia, bronchopneumonia, and interstitial pneumonia. Bacterial, community-acquired pneumonia classically show airspace consolidation of one lung segmental lobe, which is known as lobar pneumonia [7]. However, findings may vary, and other patterns are common in different types of pneumonia. Detecting pneumonia from chest radiography is a challenging task since radiologic findings may be absent or subtle in the early stages of the disease and a high prevalence of pulmonary conditions can mimic pneumonia [8]. Furthermore, some studies showed that errors are common in the interpretation of chest radiographs, due to inter-observer variation [9]. The limitation of human expert-based diagnosis has provided a strong motivation for the use of computer technology to improve the speed and accuracy of the detection process. Now, with the advent of digital CXR, there is renewed interest in using CXR interpreted by computer-aided diagnosis (CAD) software programs for the diagnosis of pneumonia [10–12]. Recent advance of deep learning algorithm for CAD on CXR, diagnosis of pneumonia is expected to improve physician`s performance for diagnosing pneumonia [13, 14].

The purpose of this study was to develop an artificial intelligence (AI) model for the diagnosis of pneumonia using the public open database of CXR and to evaluate the diagnostic accuracy of the model using an external dataset from a tertiary hospital in Korea.

## Materials and methods

### Data source

In this study, data collected from the National Institutes of Health (NIH), the Korean National Tuberculosis Association (KNTA), and a tertiary university hospital (Gachon University Gil Medical Center GUGMC, Incheon, South Korea) were used. The summary of the demographic information on the data are provided in Table 1 and S1 Data.

**Table 1:** Dataset for model development and evaluation.

	**Dataset for model development**		**Test dataset**
CXR images according to data source (normal vs. pneumonia)	n = 157,016 (120,722 vs. 36,294) from NIH (60,361 vs. 36,294) from KNTA (60,361 vs. 0)		normal: NIH(n = 106), KNTA (n = 106) pneumonia from GUGMC (n = 212)
	**Training dataset (70%)**	**Validation dataset (30%)**	
	n = 109,912 (84,506 vs. 25,406)	n = 47,104 (36,216 vs. 10,888)	n = 424 (212 vs. 212)
Number of patients (male vs. female)	62,703 (32,065 vs. 30,638)	28,463 (14,746 vs. 13,717)	424 (288 vs. 136)
Age (mean ± SD)	47 ± 16	47 ± 16	54 ± 13

Abbreviations: CXR, chest X-ray; NIH, National Institutes of Health; KNTA, Korean National Tuberculosis Association; GUGMC, Gachon University Gil Medical Center.

The institutional review board of GUGMC approved this retrospective study and waived the requirement for informed patient consent (approval number: GBIRB-2019-337).

### Dataset for model development

For model development, dataset was extracted from large-scale public open dataset of the National Institutes of Health (NIH), containing approximately 112,000 CXR images (portable network graphics, png format) of multiple labels using natural language processing (NLP) [15]; The CXR labels of abnormal data are as follows; atelectasis, cardiomegaly, consolidation, edema, effusion, emphysema, fibrosis, infiltration, mass, nodule, pleural thickening pneumonia, pneumothorax, and hernia. Along with normal CXR (n = 60,361), pneumonia (n = 36,294) was chosen based on having one or more of the following CXR labels; consolidation, effusion, infiltration, nodule, and pneumonia.

The Korean National Tuberculosis Association (KNTA) dataset consists of CXRs (DICOM format) for tuberculosis screening by KNTA and has been annotated by multiple radiologists for the presence of tuberculosis abnormalities. To allow the pneumonia model to learn various types of data, an equal number of normal data (n = 60,361) from KNTA were randomly sampled.

A total of 70% was used for model training and the remaining 30% dataset was used as a validation dataset.

### Dataset for evaluation

The test dataset was extracted from a pneumonia cohort (between January and June 2018) of a tertiary university hospital (GUGMC) as DICOM files (n = 212). The diagnosis based on the 10^th^ revision of the International Statistical Classification of Diseases and Related Health Problems (ICD-10) codes and the initial CXR was collected at the time of admission. Normal data were randomly extracted from NIH and KNTA (212 pieces of normal data, n = 106 for each) to set the ratio of test dataset between pneumonia and normal as 1:1. In addition, the longitudinal response was evaluated when the follow-up CXR became available on the 7-day after initial CXR (FU CXR) from a subset of pneumonia patients (n = 100). On FU CXR, the cases were determined as improved cases and aggravated cases using medical record based on the clinical presentation (deterioration of clinical symptom and required ventilator care and ICU transfer) and on the radiologic changes.

### Pre-processing module

The pre-processing module consists of Invert Checker, Lung Finder, and Data Rescaler. Since the file format and data type of CXR are different, the image input is put through the pre-processing module.

Invert Checker checks whether the input image is inverted in black-white format. The invert check model is a simple convolutional neural network (CNN) model designed to classify whether the image is inverted to black-white format. For the training data, 1,000 randomly extracted data from NIH and KNTA were used and the images were composed of black-white inverted images and non-inverted images on a 1:1 ratio. The Invert Check Model consists of one input layer, three hidden layers, and one output layer. The size of the input shape at the input layer is 64 × 64, the hidden layer has 128, 64, and 32 nodes respectively, and the Rectified Linear Unit (ReLU) [16] is used as an activation function. The output layer has a value between 0 and 1, and softmax [17] is used as the activation function.

Lung Finder sets the lung area as the region of interest (ROI) in the CXR data using the lung segmented image. It can minimize the learning areas other than lungs in pneumonia models. For lung segmentation, one of the artificial neural networks, U-net was used [18, 19]. The training data used were the Montgomery and Japanese Society of Radiological Technology (JSRT) datasets [20]. The lung segmentation model consists of three down-sampling layers and an up-sampling layer. The size of both the input and output shapes is 256 × 256. In the results of the lung segmentation model, parts smaller than the stated size are removed by post-processing and the lungs are cropped to the ROI.

The Data Rescaler resizes, normalizes, and standardizes the data input size to fit the input layer in the pneumonia model. The data processed by the Data Rescaler is used as training or test data in the pneumonia model.

**Fig 1:**
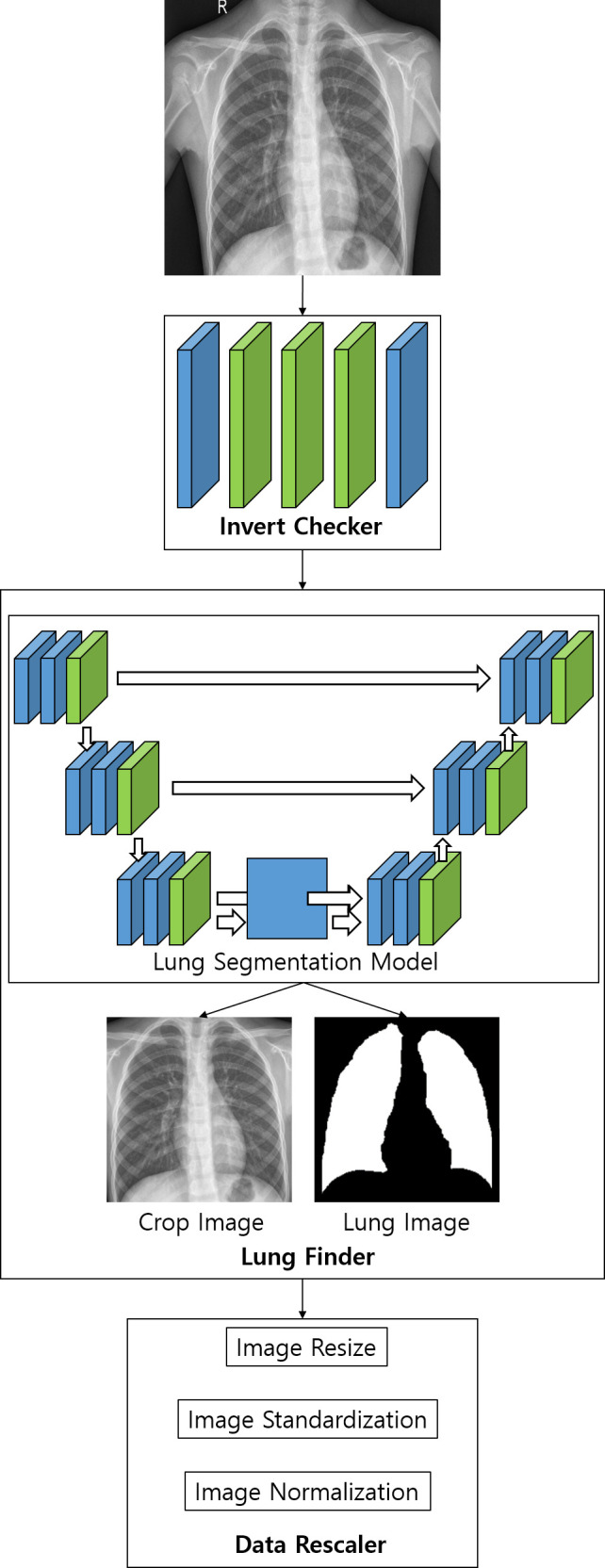
Flow diagram of the pre-processing module process.

Fig 1 shows the process flow of the pre-processing module used in this study. The data processed in the pre-processing module is used as input data for the pneumonia model.

### Classification module

**Fig 2:**
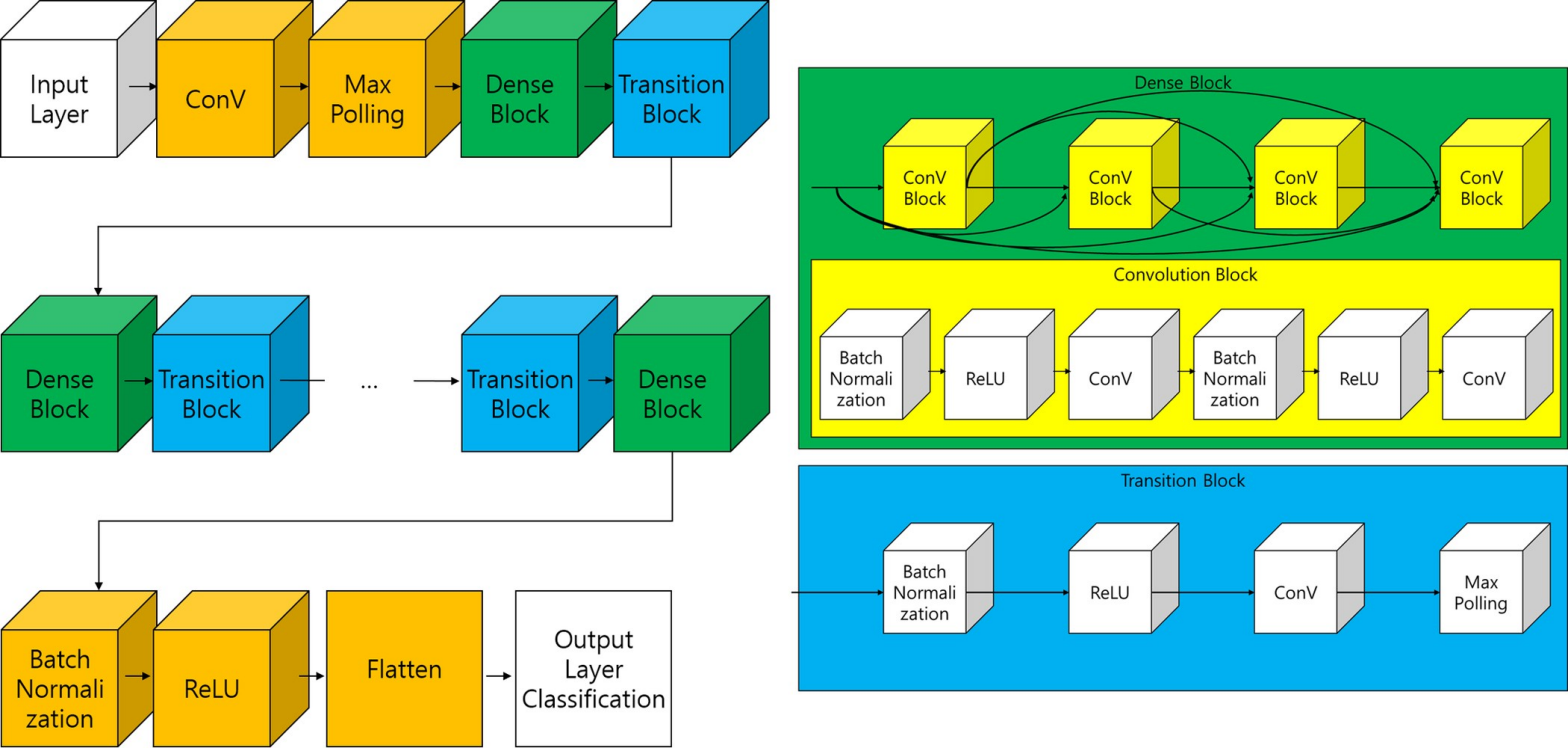
Architecture of DenseNet.

The pneumonia model is based on DenseNet [21]. DenseNet has a structure in which some layers are bundled into dense blocks and then the dense blocks are repeated. It allows the information of the feature map at the input layer to be maintained even when the AI model is deep. The basic architecture of DenseNet is shown in Fig 2.

In this study, DenseNet was used to combine datasets and ten models were trained; a summary of each model and the data augmentation used are shown in Table 2.

**Table 2:** Summary of AI models for the diagnosis of pneumonia.

Model No.	Description	Data Augmentation
Model 1	Model trained with NIH dataset	Flip(Vertical, Horizontal)
		Rotation(90, 180, 270)
Model 2	Model trained with NIH dataset	Flip(Vertical, Horizontal)
		Rotation(90, 180, 270)
		Histogram equalization
Model 3	Model trained with NIH and KNTA dataset	Flip(Vertical, Horizontal)
		Rotation(90, 180, 270)
Model 4	Model trained with NIH and KNTA dataset	Flip(Vertical, Horizontal)
		Rotation(90, 180, 270)
		Histogram equalization
Model 5	Model trained with NIH dataset first and transfer learning KNTA dataset (using Model 1)	Flip(Vertical, Horizontal)
		Rotation(90, 180, 270)
		Invert
Model 6	Model trained with NIH dataset first and transfer learning KNTA dataset (using Model 1)	Flip(Vertical, Horizontal)
		Rotation(90, 180, 270)
		Histogram equalization
		Invert
Model 7	Model trained with NIH dataset (abnormal: pneumonia, effusion, consolidation)	Flip(Vertical, Horizontal)
		Rotation(90, 180, 270)
		Invert
Model 8	Model trained with NIH dataset (abnormal: pneumonia, effusion, consolidation)	Flip(Vertical, Horizontal)
		Rotation(90, 180, 270)
		Histogram equalization
		Invert
Model 9	Model trained with NIH dataset first and transfer learning KNTA dataset (using Model 1)	Flip(Vertical, Horizontal)
		Rotation(90, 180, 270, -15 to 15)
		Invert
Model 10	Model trained with NIH dataset first and transfer learning KNTA dataset (using Model 1)	Flip(Vertical, Horizontal)
		Rotation(90, 180, 270, -15 to 15)
		Histogram equalization
		Invert

The performance of the AI models was quantitatively compared using sensitivity, specificity, and accuracy. In addition, to find the optimal model, each model was combined with others to create and to be used an ensemble model. Since the ensemble model uses a combination of several models, it has the advantage of improving performance as well as reducing the effect of overfitting. The number of gadgets of the ensemble model can be obtained by combining the models. For this study, the number equals to $\sum_{m=1}^{10}{}_{10}C_{m}$, a total of 1,023 combinations. The probability score of the ensemble model was obtained by using the RMS (Root Mean Square) method on the probability score of the models composing the ensemble model.

The optimal ensemble model is obtained by sorting the area under the receiver operating characteristic curve (AUC) [22] for the entire combination in descending order, where the top 20 combinations are selected, based on sensitivity and specificity. Since the CXR is regarded as an initial simple assessment method for pneumonia diagnosis, we set the sensitivity to outperform the specificity for the pneumonia model. Therefore, the sensitivity is multiplied by a weight of 0.7 and specificity is multiplied by 0.3. The optimal ensemble model is established by sorting it in descending order based on this score.

### Statistical analysis

Results are presented as a percentage for categorical variables and as a mean (± standard deviation) for continuous variables. The diagnostic performance of AI models for pneumonia was compared using test dataset, in terms of sensitivity, specificity, and accuracy, to enable classification as pneumonia or not. Moreover, the AUC for each model was also calculated to evaluate general classification performance.

To evaluate the probability score of the AI pneumonia model that could represent the longitudinal change of pneumonia, the change of probability score (Δ = probability score on FU CXR − probability score on baseline CXR) was compared using the paired sample T-test between improved cases and aggravated cases on CXR taken at the follow-up, 7 days after the initial diagnosis.

The open-source statistical software Python version 3.6.5 was used to analyze and evaluate the AI model and the diagnostic performance. *P*-values of less than 0.05 (two-sided) were considered significant.

## Results

Table 3 shows the results of the performance of variable AI models for the diagnosis of pneumonia with external test dataset. AUC, sensitivity, and specificity are used as performance evaluation indicators. Table 3 shows that the AUC of Model 1 was 0.973 and the sensitivity was 0.972, which was also the highest. However, the specificity shows a low value of 0.764, which is difficult to use as a pneumonia model. Therefore, in this study, the optimal ensemble model combination was found by combining each model. The ensemble model compared and analyzed the performance of the values obtained by calculating the probability, which is the result of each model, with the probability of another model that does not allow duplication. The ensemble model’s result is calculated by the RMS method. AUC, which is not affected by the threshold, was used as a performance indicator, and through this, a combination of ensemble models for optimal pneumonia diagnosis was found. The results are shown in Table 4, which shows the top three combination ensemble models with high AUC for the diagnosis of pneumonia.

**Table 3:** Performance of AI models for pneumonia.

Model Number	AUC	Sensitivity	Specificity
Model 1	0.973	0.972	0.764
Model 2	0.311	0.873	0.024
Model 3	0.630	0.802	0.425
Model 4	0.960	0.646	0.995
Model 5	0.726	0.490	0.962
Model 6	0.711	0.448	0.975
Model 7	0.665	0.412	0.914
Model 8	0.623	0.310	0.935
Model 9	0.723	0.624	0.822
Model 10	0.641	0.297	0.987

Abbreviations: AUC, area under the receiver operating characteristic curve.

**Table 4:** Performance of the ensemble models.

Used Model Number	AUC	Sensitivity	Specificity
Model 1 and Model 4	0.983	0.962	0.901
Model 1, Model 4, and Model 5	0.982	0.939	0.929
Model 1, Model 4, Model 5, and Model 10	0.981	0.962	0.885

Abbreviations: AUC, area under the receiver operating characteristic curve.

**Fig 3:**
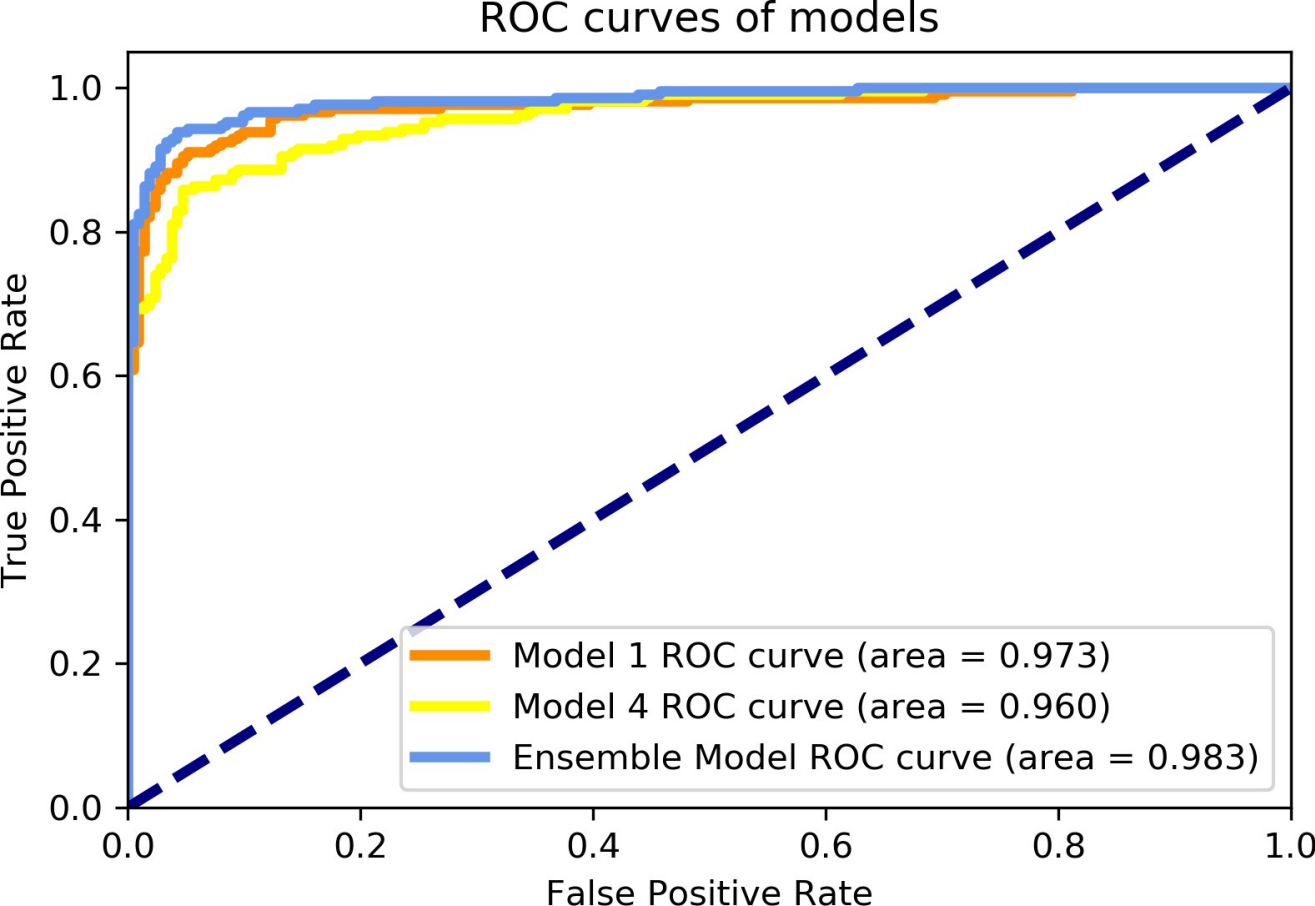
ROC curves of AI models for pneumonia.

It was confirmed that the optimal ensemble model occurs when Models 1 and 4 are combined. The receiver operating characteristic (ROC) curves on the graphs of Models 1 and 4 and the ensemble models (Model 1 and Model 4) are shown in Fig 3.

**Fig 4:**
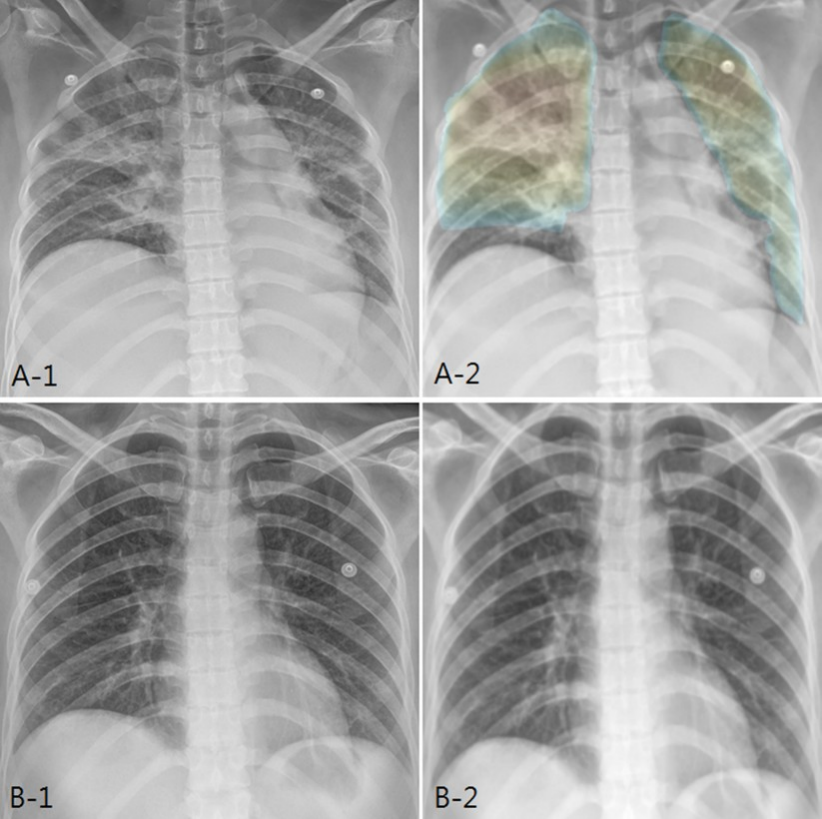
AI probability score changes and heatmap in improved patient. (a) In a 42-year-old female patient, initial chest X-ray shows bilateral patchy increased opacity, compatible with pneumonia. AI pneumonia model (A-2) shows the probability score of 0.787 and color map for pneumonia. (b) After 7 days of treatment, the chest X-ray shows marked improvement. AI pneumonia model (B-2) shows the decrease of probability score (0.318) for pneumonia.

**Fig 5:**
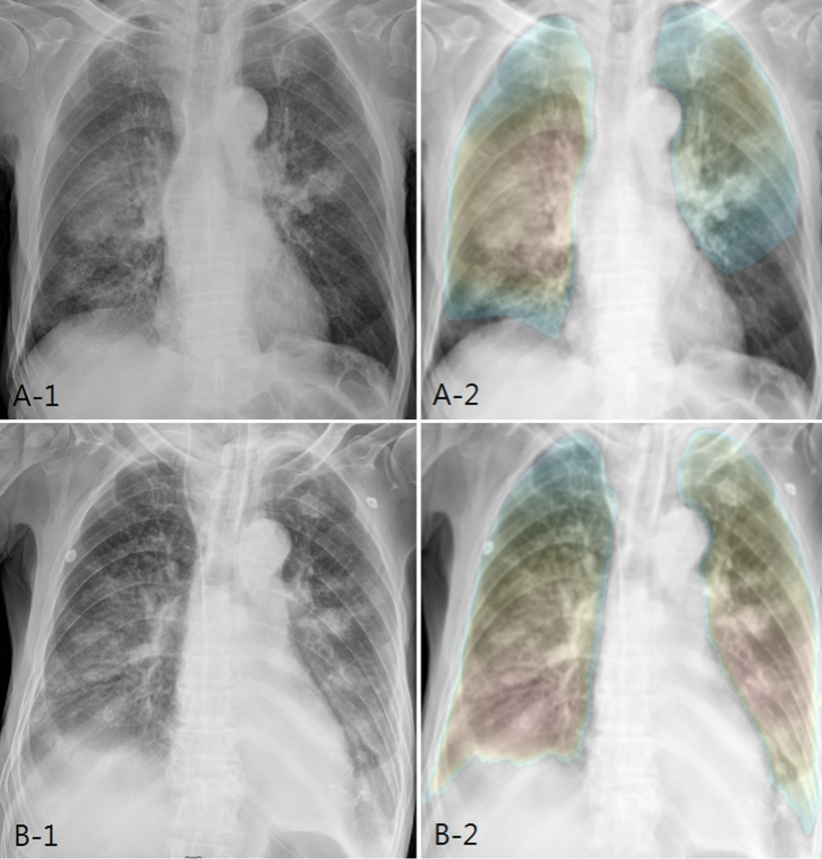
AI probability score changes and heatmap in aggravated patient. (a) In an 81-year-old male patient, initial chest X-ray shows bilateral patchy increased opacity, compatible with pneumonia. AI pneumonia model (A-2) shows the probability score of 0.833 and color map for pneumonia. (b) After 7 days of treatment, the chest X-ray shows slight aggravation. AI pneumonia model (B-2) shows slight increase of probability score (0.876) and increased extent of color map for pneumonia.

In addition, the paired sample T-test was performed to evaluate whether the probability score of the AI pneumonia model represents the longitudinal change of pneumonia. Using a follow-up CXR (CXR Day 8) taken seven days after the diagnosis of pneumonia (n = 100) with the original test dataset (CXR Day 1), the patients showed either improvement (n = 85) or aggravation (n = 15) based on the clinical progression and radiologic change (Figs 4 and 5).

**Fig 6:**
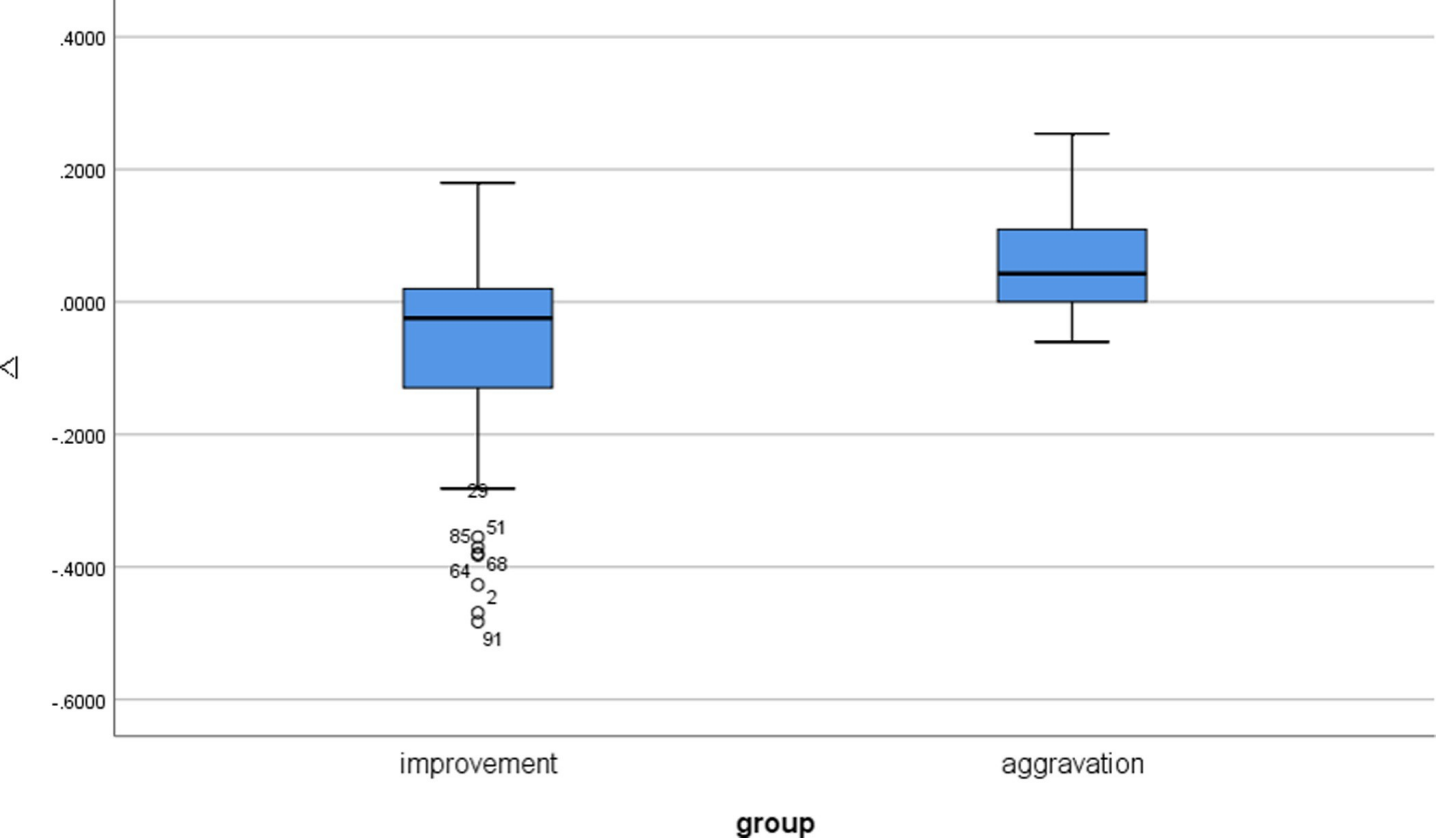
Box plot of probability score changes on follow-up images on pneumonia test dataset (n = 100).

The probability score change showed a significant difference between improvement cases and aggravation cases (Δ = -0.06 ± 0.14 vs. 0.06 ± 0.09, for improved cases vs. aggravated cases. *P* = 0.001) (Fig 6).

## Discussion

The technical advance in artificial intelligence (AI) offers rapid and accurate automated diagnosis in medicine. Deep learning algorithm showed excellent performance diagnosis of CXR in lung cancer, pneumonia, tuberculosis, and multiple abnormal findings [13, 14, 23–25]. In a study of deep-learning based neural network algorithm for major thoracic diseases including pneumonia on CXR, the algorithm demonstrated significantly higher performance than that of the physician groups (AUC, 0.983 vs 0.814–0.932; all *P* < .005) and significant improvements (0.814–0.932 to 0.904–0.958; all *P* < .005) in performance were observed in physician groups with the assistance of the algorithm [13].

The CXR is one of the most frequently performed procedures in medicine and the first imaging modality for diagnosis of pneumonia. The main role is in the detection or exclusion of the presence of pneumonia. Other roles include the narrowing down of differential diagnosis of pneumonia with regards to the causative organisms involved. Furthermore, the response to antibiotic therapy could be assessed by evaluating follow-up imaging studies. However, the reading of CXR is also one of the most complex radiology tasks; it represents a huge workload in all institutions and some countries have very few radiologists to interpret CXR. Furthermore, detection errors are common due to the low contrast between lesions and non-lesion regions such as the surrounding lung and the superposition of bone/mediastinal structures. Computer-assisted tools can be helpful in the reading of CXR. The CAD system has been reported to improve reader accuracy for the detection of lung lesions previously missed on CXR [26]. AI algorithms derived from CXR would also be of great benefit to undertake massive screening programs that could take place in any country with access to X-ray equipment and to support the diagnosis of pneumonia. Since CXR is used across the world due to its affordability, including those areas with few or no radiologists, AI can be applied to facilitate automated screening for pneumonia even in a low-resource setting.

In this study, AI model for pneumonia on CXR was designed and developed. For the developed AI models, not only the performance of single models but also the performance of the ensemble model was tested. As a result, the AUC of 0.973, the highest performance among the single models, was increased to AUC of 0.983 through the ensemble modelling for the diagnosis of pneumonia with the test dataset from a completely different data source to the training data.

Furthermore, the probability score change on FU CXR can represent the clinical course of the pneumonia. One of main role of imaging is to evaluate the response to antibiotic therapy by observing follow-up imaging studies. Thus, we evaluated the AI probability score on a follow-up CXR taken 7 days after the diagnosis of pneumonia. The conventional 10- to 14-day treatment duration anecdotal patterns of behavior is not supported by current evidence. Recent guidelines recommend 5 to 7 days of treatment based on studies showing that a period shorter than 7 days was as effective as longer duration of therapy for mild to moderate community-acquired pneumonia [27]. These suggest the possible application of screening and quantitative analysis to identify patients at risk of poor clinical course.

To evaluate the severity of infection and monitoring of the disease, imaging studies play an important role. The AI-based pneumonia model on CXR images can be one of the fastest and the most quantitative image-based triage tools for diagnosis and treatment monitoring with the fully automated image analysis function to effectively control a large number of suspected or infected patients. A more accurate analysis can be provided by combining the patient’s information such as fever, respiratory condition, and underlying conditions.

The present study has several limitations. First, we trained the AI model for pneumonia from NIH and KNTA dataset; the annotation methods were heterogeneous and were not intentionally for pneumonia. The NIH dataset was loosely annotated using NLP and text mining method with several abnormal findings under various clinical settings. The KNTA dataset was annotated by radiologists for the existence of tuberculosis lesion under tuberculous screening setting. Second, we evaluated the diagnostic performance using external dataset of pneumonia cohort from a tertiary university hospital from Korea. Moreover, the normal data of the test dataset, extracted from KNTA and NIH, were not used for training. Finally, we utilized and validated with data solely from Korea and it does not guarantee the same diagnostic performance with other populations of different nationalities, races, and clinical settings.

In conclusion, our study developed an AI pneumonia model on CXR and demonstrates good performance for the diagnosis and follow-up monitoring of pneumonia. There is likely a promising role of the AI pneumonia model as an effective screening method for pneumonia at the primary care level in settings where access to radiologists is limited. Further study is warranted to evaluate the usefulness of the AI model for pneumonia in the diagnosis and monitoring of treatment.

## Supporting information

S1 Data (XLSX)
